# Effects of allopurinol on renal function in patients with diabetes: a systematic review and meta-analysis

**DOI:** 10.1080/0886022X.2022.2068443

**Published:** 2022-07-20

**Authors:** Qian Luo, Yuzi Cai, Qihan Zhao, Lei Tian, Yuning Liu, Wei jing Liu

**Affiliations:** a Dongzhimen Hospital Affiliated to Beijing University of Chinese Medicine, Beijing University of Chinese Medicine, Beijing, China; b Key Laboratory of Chinese Internal Medicine of Ministry of Education and Beijing, Beijing, China; c Zhanjiang Key Laboratory of Prevention and Management of Chronic Kidney Disease, Guangdong Medical University, Zhanjiang, Guangdong, China

**Keywords:** Diabetes mellitus, hyperuricemia, allopurinol, meta-analysis, renal function

## Abstract

**Background/Objective:**

Diabetes mellitus is a common “non-gout” disease with high incidence. Several studies have shown that serum uric acid level in patients with diabetes is higher than that in healthy individuals, and is accompanied by severe albuminuria and high serum creatinine (Scr). Recent clinical studies have found that uric acid-lowering therapy (such as allopurinol) could reduce urinary albumin excretion rates (UAER) and Scr, increase eGFR, and thus reduce kidney damage in patients with diabetes. Therefore, this meta-analysis [PROSPERO CRD42021274465] intended to evaluate the efficacy and safety of allopurinol in patients with diabetes mellitus.

**Methods:**

We thoroughly searched five electronic resource databases for randomized controlled trials (RCTs) that compared the efficacy and safety of allopurinol versus conventional treatment or placebo for the treatment of patients with diabetes mellitus. Predetermined outcomes were considered continuous variables, mean difference (MD) was used for the determination of effect size (standardized mean difference [SMD] was used to determine the effect size when there were different evaluation criteria in different articles), and the corresponding 95% confidence interval (CI) was calculated. All outcome measures were analyzed using a random-effects model for data analysis.

**Results:**

Ten eligible trials with a total of 866 participants were included in the meta-analysis. Allopurinol was more effective in decreasing serum uric acid (SUA) levels compared with conventional treatment (*p* = 0.0001) or placebo (*p* < 0.00001). Moreover, the levels of 24-hour urine protein were significantly lower in the allopurinol group (*p* < 0.00001). The subgroup analysis of Scr showed that the Scr of patients with an allopurinol treatment duration of fewer than six months was significantly lower than that of the control group (*p* = 0.03). No significant difference in adverse events (AEs) was identified between the treatment and control groups.

**Conclusions:**

Our meta-analysis of RCTs showed that oral administration of allopurinol effectively reduced SUA levels in patients with diabetes, and patients’ renal function was protected. More RCTs with larger sample sizes and higher quality are needed to clarify the role of allopurinol use in decreasing blood pressure, maintaining blood glucose levels, and improving renal function in patients with diabetes.

## Introduction

Hyperuricemia (HUA) is defined as a serum uric acid level >420 μmol/L in men and >360 μmol/L in women. Its main clinical consequence is gout with or without deposition [1]. Over the last few decades, the prevalence of hyperuricemia has risen in many countries. Hyperuricemia has been proven to be closely associated with the global increase in several “non-gout” diseases, such as hypertension, obesity, metabolic syndrome, type 2 diabetes mellitus, and chronic kidney disease (CKD) [2]. Diabetes mellitus is a common “non-gout” disease with a high incidence. Epidemiological data have shown that with every 1 mg/dL increase in serum uric acid level in patients with type 1 diabetes, the risk of diabetic kidney disease (DKD) increases by 80% [3]. Fouad et al. [4] found that the serum uric acid level of type 2 diabetes mellitus (T2DM) patients was significantly higher than that of individuals without diabetes, and HUA could accelerate the occurrence of DKD in T2DM patients. In addition, it has been shown that serum uric acid levels are strong and independent predictors of estimated glomerular filtration rate (eGFR) decline and albuminuria in a study including patients with diabetes [5–7]. Although the American College of Rheumatology (ACR) [8] has not defined the causal role of urates in “non-gout” diseases, recent clinical studies have shown that urate-lowering treatment can decrease urinary albumin excretion rate (UAER) and Scr, increase eGFR, and reduce the severity of proteinuria in patients with diabetes; thus, urate-lowering treatment may become an adjuvant cost-effective therapy for diabetes mellitus [1,2,5].

Allopurinol, which belongs to xanthine oxidase inhibitors, is one of the first-line urate-lowering agents used in patients with gout. Allopurinol inhibits purine synthesis and decreases uric acid formation [8,9]. The 2020 ACR guideline strongly recommends allopurinol as the first-line therapy, especially for patients with moderate-to-severe CKD (CKD stage 3 or worse). In contrast to the 2012 ACR guidelines, the preference for allopurinol in the 2020 guidelines is based in part on the cost of each medication [8]. Moreover, some studies have shown that allopurinol has potentially greater cardiovascular safety than febuxostat [8]. Therefore, it is necessary to conduct a meta-analysis to evaluate the efficacy and safety of allopurinol on renal function in patients with diabetes mellitus. Last year, there was a retrospective analysis on the topic [10], which was limited to older adults (>65 years old) and had a small sample size and limited generalizability. Here, we conducted a meta-analysis of randomized controlled trials (RCTs) with the aim to clarify the role of allopurinol in decreasing blood pressure, maintaining blood glucose levels, and improving renal function in patients with diabetes.

## Materials and methods

### Search strategy

We comprehensively searched PubMed, Embase, Cochrane Library, SinoMed, and China National Knowledge Infrastructure from inception until August 2021 for RCTs that had investigated allopurinol for renal function in patients with diabetes. Additional studies were searched in the reference lists of all identified publications, including relevant meta-analyses and systematic reviews.

### Inclusion and exclusion criteria

We included clinical studies that satisfied the following criteria: type of study limited to RCTs; participants in the included studies were patients with diabetes mellitus; studies eligible for inclusion used allopurinol as the intervention arm (dosage was not restricted); the studies also had to contain a control arm receiving conventional treatment or placebo; and outcome indicators were (1) SUA, 24-h urine protein (24H-P), blood pressure, Scr, fasting blood glucose (FBG), homeostatic model assessment for insulin resistance (HOMA-IR), glycated hemoglobin (HbA1c), and (2) adverse effects (AEs). The exclusion criteria were as follows: the participant’s disease is not diabetes mellitus; both the intervention and the comparator arms received allopurinol; the full text or the data of outcome indicators were unavailable.

### Study selection and data extraction

Two reviewers (QL and YC) independently extracted data from original trial reports using a standardized form. Data extracted included study characteristics (first author, publication year, sample size, intervention and control, the period of treatment), characteristics of patients (inclusion criteria, background treatments, mean age, proportion of men, baseline uric acid levels), reported outcomes (SUA, 24H-P, blood pressure, Scr, FBG, HOMA-IR, HbA1c, and AEs), and information on methodology. The risk of bias of RCTs was assessed using the Cochrane Collaboration’s tool. Two investigators (QL and YC) independently completed the assessments; discrepancies were discussed with other members (QZ and LT) and resolved by consensus.

### Statistical analysis

The data entry and analysis were done using Excel 2016 (Microsoft, Redmond, WA, USA), Stata statistical software version 12.0 (StataCorp LP, College Station, TX, USA), and ReviewManager (RevMan) software version 5.3. Predetermined outcomes were considered as continuous variables; the mean difference (MD) was used for the determination of effect size (standardized mean difference [SMD] was used to determine the effect size when there were different evaluation criteria in different articles), and the corresponding 95% confidence interval (CI) was calculated. All outcome measures were analyzed using a random-effects model for data analysis. Subgroup analysis or sensitivity analysis was conducted to explore the underlying causes of heterogeneity in treatment outcomes. The funnel plot (studies >10) was used to evaluate the publication bias for the primary outcomes.

## Results

### Study characteristics

**Figure 1.**
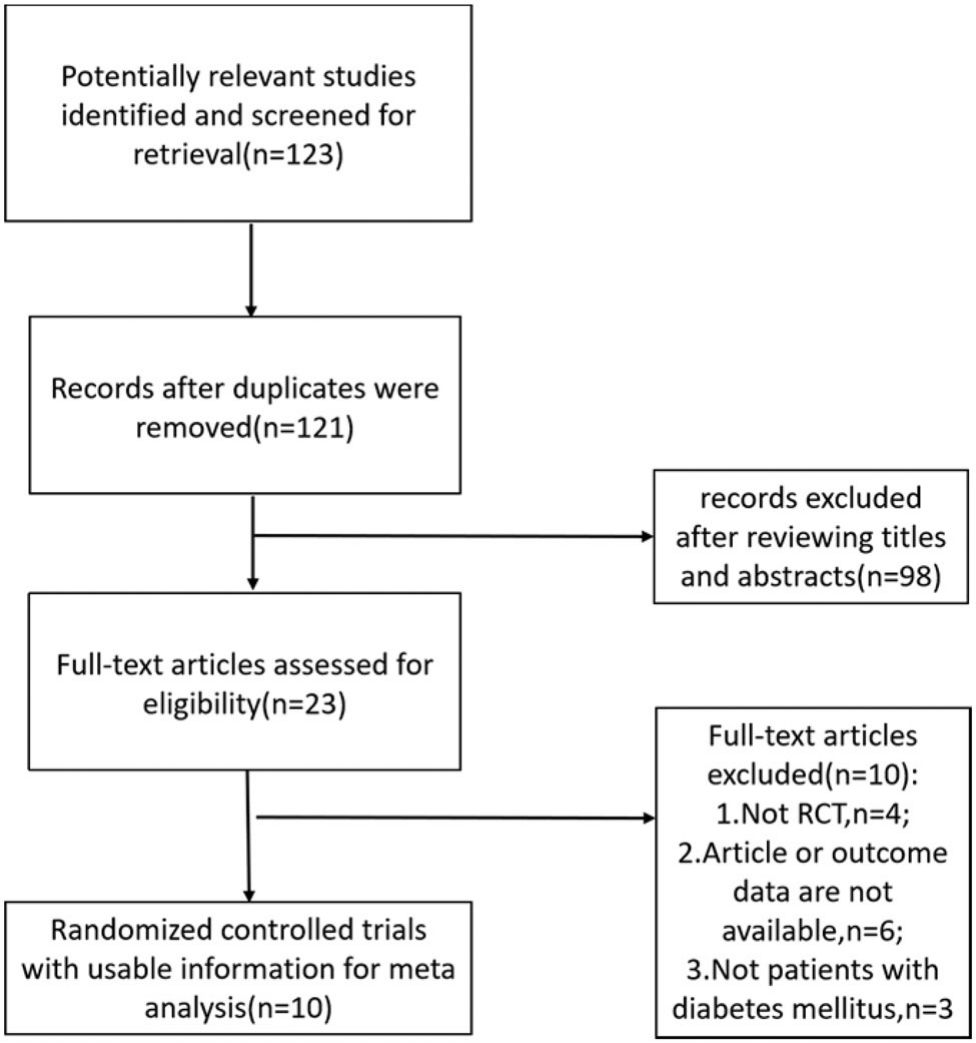
Flow chart of literature search and selection.

The search identified 123 articles, among which 12 were duplicates. Subsequently, 121 titles and abstracts were screened, leaving 23 articles for full-text screening. Finally, 10 eligible manuscripts (*n* = 866) (Figure 1) evaluated the efficacy and safety of allopurinol for renal function in patients with diabetes mellitus. Figure 1 shows the screening process. Table 1 presents the main characteristics of the included trials.

**Table 1.** Characteristics of the included studies in the meta-analysis.

Study	Inclusion criteria	Baseline uric acid (μmol/L)	No. of patient (% of male)	Age, (yr) mean ± SD	Treatment Duration	Intervention
Peng Liu [11]	T2DM with UAER < 20 μg/min; SUA 420–476 µmol/L	I:433 ± 11 C:432 ± 9	I:82(46.3%) C:70(45.7%)	I:50 ± 10 C:51 ± 11	36 months	I: 100 mg Allopurinol/day (to reach SU*A* < 360μmol/L) C: Conventional Treatment
Pilemann-L [12]	T1DM with SU*A* ≥ 260μmol/L； UACR ≥ 30 mg/g； eGFR ≥ 40 ml/min/1.73 m2	I:343 ± 100 C:343 ± 100	I:26(73%) C:26(73%)	I:59 ± 10 C:59 ± 10	2 months	I: 400 mg Allopurinol/day C: placebo
AliMomeni [13]	T2DM with 24 h-*p* ≥ 500mg/24h; Scr < 265.2 μmol/L	I:354.5 ± 71.9 C:386.6 ± 130.8	I:20(82%) C:20(82%)	I:56.3 ± 10.6 C:59.1 ± 10.6	4 months	I: 100 mg Allopurinol/day C: placebo
Tang [14]	T2DM； Male ≥ 420μmol/L Female ≥ 360μmol/L	I:448.3 ± 55.7 C:452.5 ± 51.6	I:40(67%) C:40(67%)	I:55.6 ± 12.8 C:55.6 ± 12.8	3 months	I: 300 mg Allopurinol/day C: Conventional Treatment
He [15]	DM with UACR > 300 mg/g； Male > 420μmol/L Female > 360μmol/L	I:460 ± 73 C:450 ± 132	I:80(54%) C:80(60%)	I:49.2 ± 9.8 C:48.1 ± 9.7	6 months	I: 300 mg Allopurinol/day C: placebo
Li [16]	T2DM； Scr 186–442μmol/L Male > 420μmol/L Female > 360μmol/L	I:549.72 ± 97.85 C:499.92 ± 102.74	I:29(55%) C:24(58%)	I:60.3 ± 11.7 C:56.5 ± 9.7	12 months	I:-300 mg Allopurinol/day C:Conventional Treatment
Tan [17]	T2DM； eGFR 30–60 ml/min； 24h-*p* > 0.5 g/24h； Male 420–600μmol/L Female 360–600μmol/L	I:531.23 ± 57.31 C:511.59 ± 60.32	I:72(51%) C:68(51%)	I:59.3 ± 9.2 C:58.6 ± 8.3	6 months	I: Allopurinol/day (to reach SU*A* < 360μmol/L) C: placebo
Wang [18]	DM； Male > 420μmol/L Female > 360μmol/L; Scr < 123μmol/L	I:458.85 ± 20.33 C:456.44 ± 18.55	I:27(55%) C:25(56%)	I:55.24 ± 10.05 C:54.12 ± 11.97	2 months	I:-50–100 mg Allopurinol/day C:Conventional Treatment
Chen [19]	T2DM； Male ≥ 420μmol/L Female ≥ 360μmol/L; UACR 30–300 mg/g；	I:482.01 ± 96.02 C:466.95 ± 76.21	I:31(72%) C:30(76%)	I:56.7 ± 8.5 C:56.6 ± 8.7	6 months	I:I: 50–300 mg Allopurinol/day (to reach SU*A* < 360μmol/L) C:Conventional Treatment
Peng [4]	DM； Male > 420μmol/L Female > 360μmol/L; 24h-*p* > 0.5 g/24h	I:445.5 ± 55.3 C:448.2 ± 63.6	I:39(67%) C:37(62%)	I:76.4 ± 3.2 C:77.8 ± 3.8	3 months	I:100–200 mg Allopurinol/day (to reach SU*A* < 357μmol/L) C: placebo

Search strategies: Our search included five databases including PubMed, Embase, Cochrane Library, SinoMed, China National Knowledge Infrastructure. The following term search strategy was used in the PubMed database, and appropriate modifications were made to suit other databases.

Pubmed: Search: (((((((((((((((((((((((((((((((((((((((((((((Uribenz) OR (Allopurin)) OR (Allorin)) OR (Allpargin)) OR (Allural)) OR (Pan Quimica)) OR (Apulonga)) OR (Apurin)) OR (Atisuril)) OR (Bleminol)) OR (Caplenal)) OR (Capurate)) OR (Cellidrin)) OR (Embarin)) OR (Suspendol)) OR (Foligan)) OR (Hamarin)) OR (Lopurin)) OR (Lysuron)) OR (Jenapurinol)) OR (Milurit)) OR (Milurite)) OR (Novopurol)) OR (Novopurol)) OR (Uripurinol)) OR (Urosin)) OR (Urtias)) OR (Xanthomax)) OR (Uridocid)) OR (Xanturic)) OR (Zygout)) OR (Zyloprim)) OR (Zyloric)) OR (Pureduct)) OR (Purinol)) OR (Progout)) OR (Remid)) OR (Rimapurinol)) OR (Roucol)) OR (Tipuric)) OR (Allohexal)) OR (Allohexan)) OR (Alloprin)) OR ("Allopurinol"[Mesh]))) AND ((((((((Diabetes Insipidus) OR (Diet, Diabetic)) OR (Prediabetic State)) OR (Scleredema Adultorum)) OR (Glycation End Products, Advanced)) OR (Glucose Intolerance)) OR (Gastroparesis)) OR ("Diabetes Mellitus"[Mesh])) Sort by: Publication Date.

### Evaluation of the risk of bias of the selected studies

**Figure 2.**
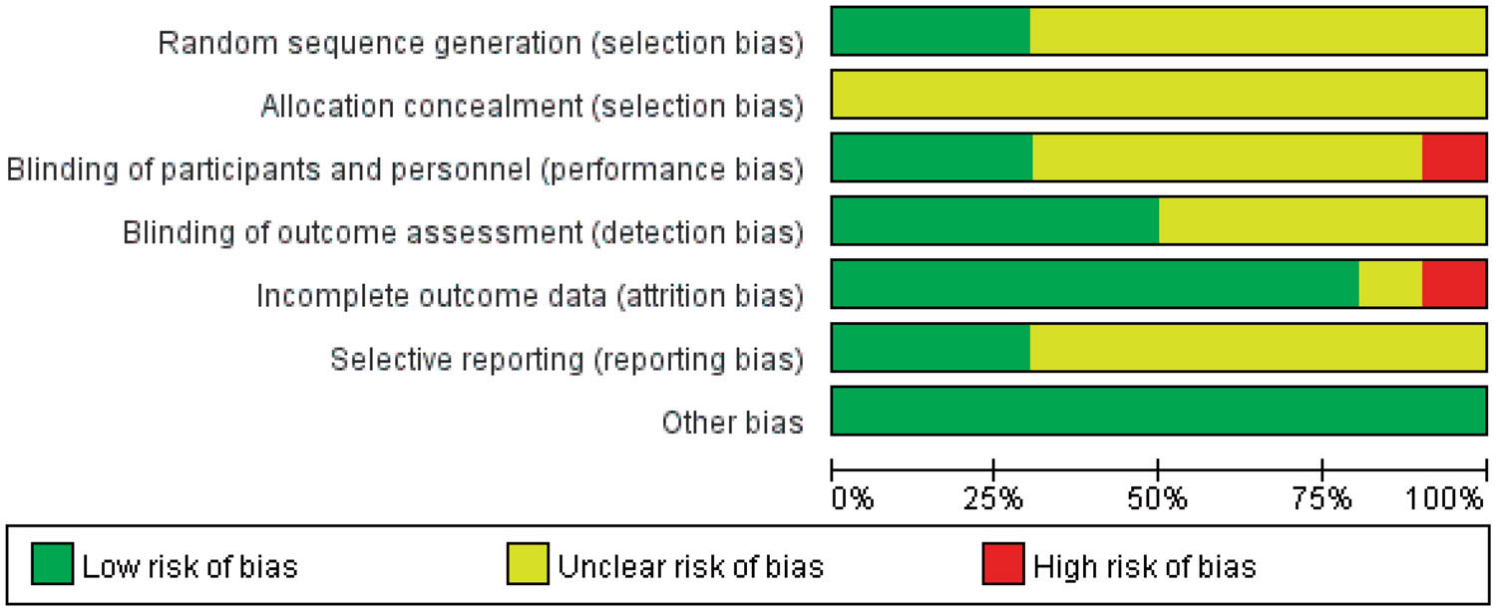
Risk-of-bias summary using the Cochrane Risk-of-Bias tool.

**Figure 3.**
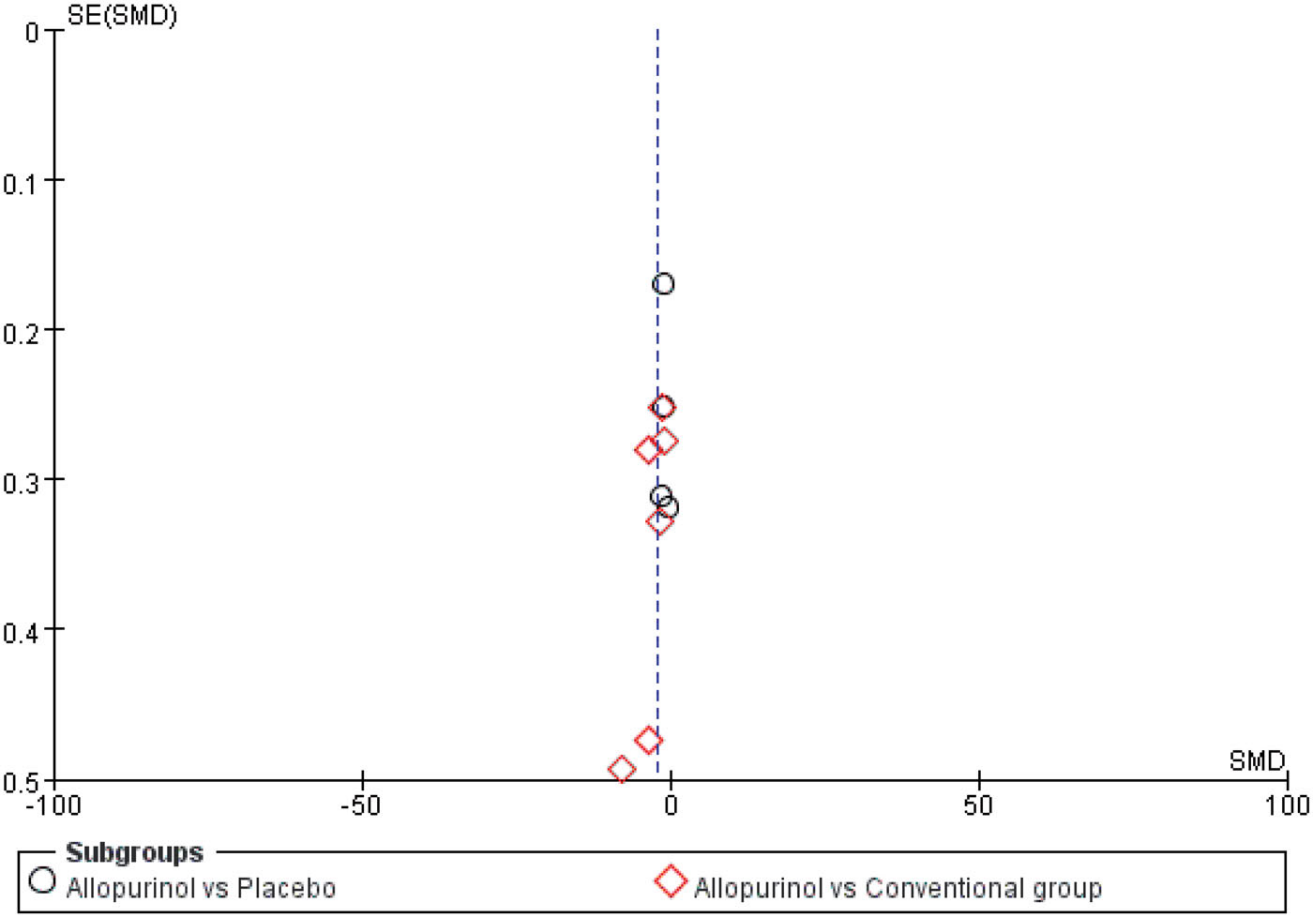
The funnel plot for SUA.

The risk of bias for the included RCTs was assessed using the Cochrane Risk-of-Bias tool. All of the studies had an unclear risk of bias for allocation concealment, considering that detailed information was not provided. Moreover, most RCTs had an unclear risk of bias for sequence generation, blinding of the outcome, and selective reporting. However, one study [17] had a high risk of bias for incomplete outcome data due to the loss of participants for AEs. Liu’s study [11] had a high risk of bias for blinding participants and personnel because they used the open-label method in designing the study. As for other types of bias, all were judged to be at low risk. The risk-of-bias assessment of the included trials is shown in Figure 2. In addition, the funnel plot indicated that publication bias did not affect the stability of SUA (Figure 3).

#### Changes in SUA

**Figure 4.**
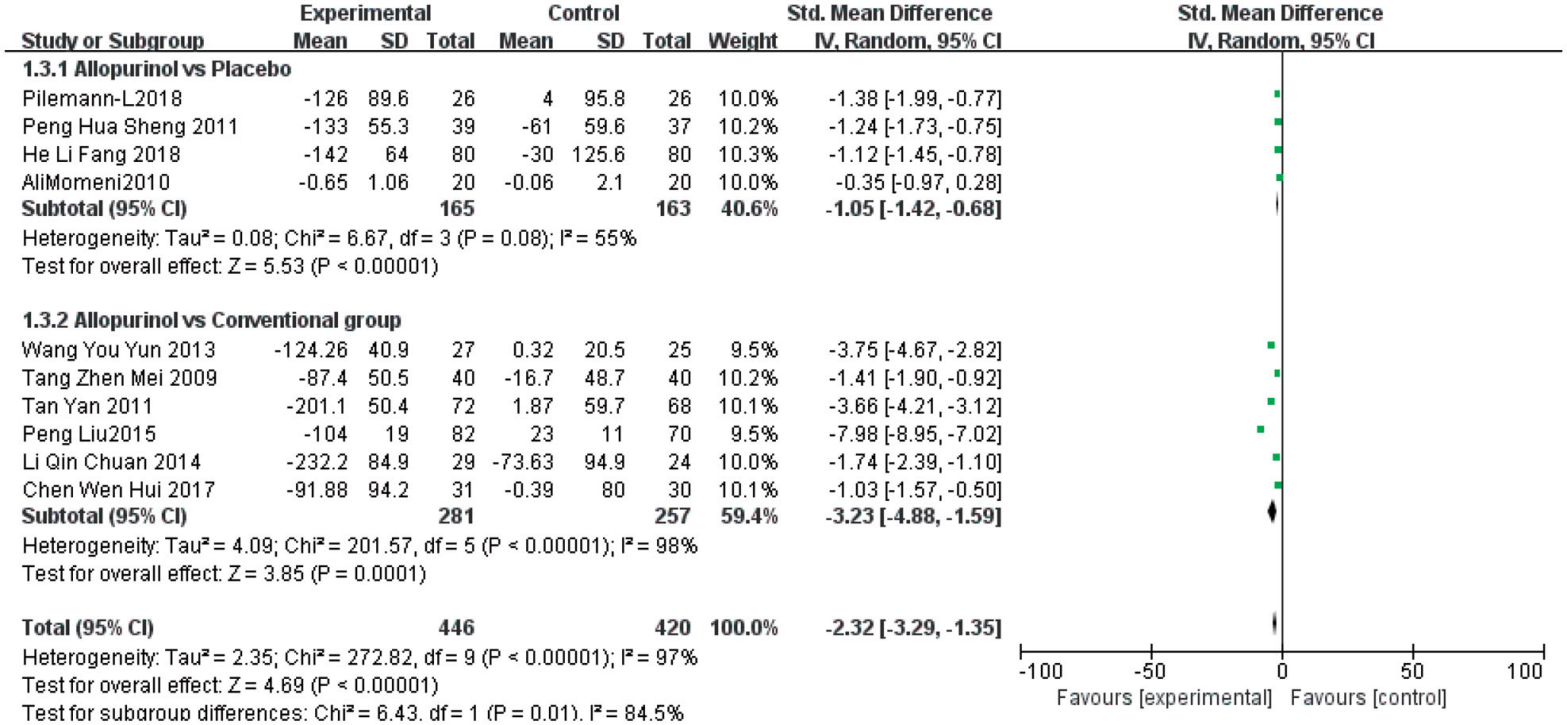
The meta-analysis results of Allopurinol for SUA.

All RCTs [3,4,12–19] investigated the change in serum uric acid level of the treatment group (*n* = 446) versus the control group (*n* = 420). As shown in Figure 4, allopurinol decreased serum uric acid more effectively than conventional treatment did (SMD, −3.23; 95% CI, −4.88 to −1.59; *I*^2^, 98%; *p* = 0.0001) or placebo (SMD, −1.05; 95% CI, −1.42 to −0.68; *I*^2^, 55%; *p* < 0.00001) (the Forest plot in Figure 4).

#### Changes in Scr and 24H-P

**Figure 5.**
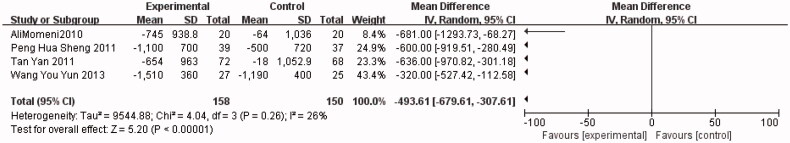
The meta-analysis results of Allopurinol for 24H-P.

Four trials [4,13,17,18] compared 24-h urine protein between allopurinol (*n* = 158) and control (*n* = 150) groups. As shown in Figure 5, 24H-P was significantly lower in the allopurinol group than in the control group (weighted mean difference (WMD), −493.61; 95% CI, −679.61 to −307.61; *I*^2^, 26%; *p* < 0.00001).

**Figure 6.**
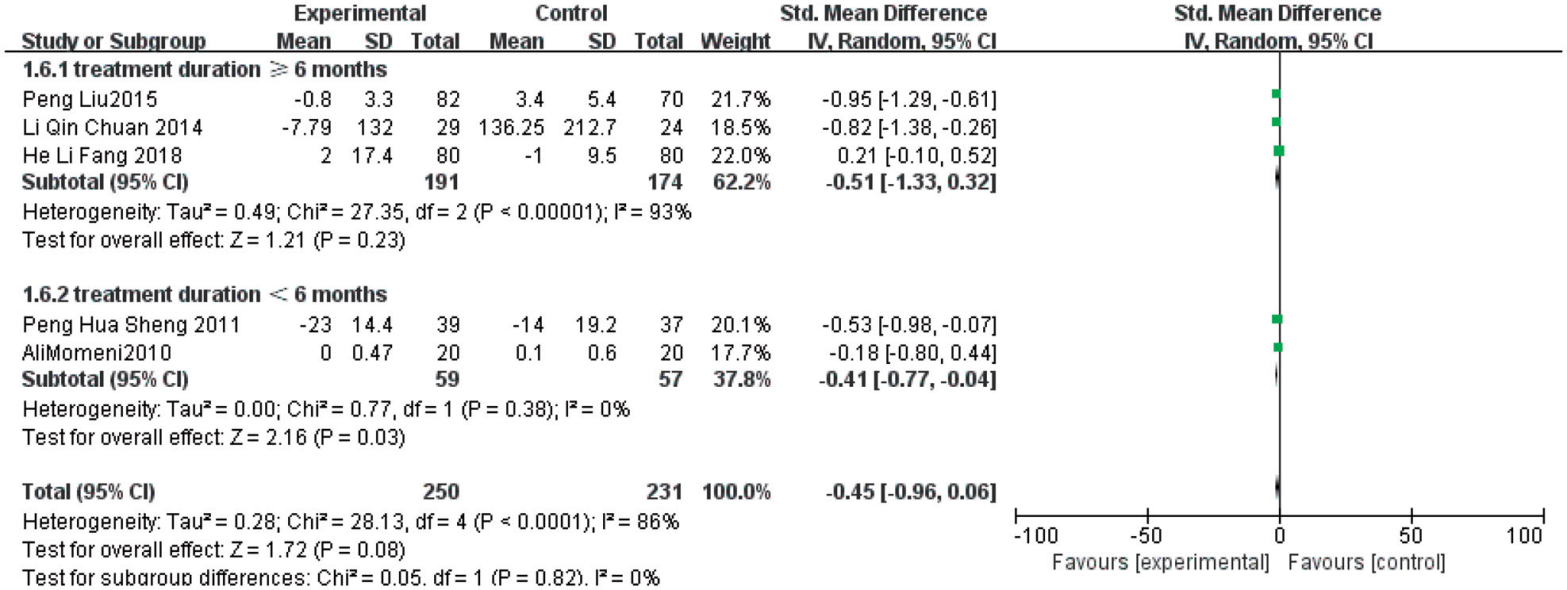
The meta-analysis results of Allopurinol for Scr.

As for Scr, five trials [4,11,13,15,16] compared the serum creatinine level between the allopurinol (*n* = 250) and control (*n* = 231) groups. No significant difference was observed between the results of the studies (SMD, −0.45; 95% CI, −0.96 to 0.06; *I*^2^, 86%; *p* = 0.08). However, a significantly lower Scr level was found in the allopurinol group after a subgroup analysis of studies with a treatment duration of fewer than six months (SMD, −0.41; 95% CI, −0.77 to −0.04; *I*^2^, 0%; *p* = 0.03) (the Forest plots from Figure 6).

#### Changes in FBG, HOMA-IR, and HbA1c

**Figure 7.**
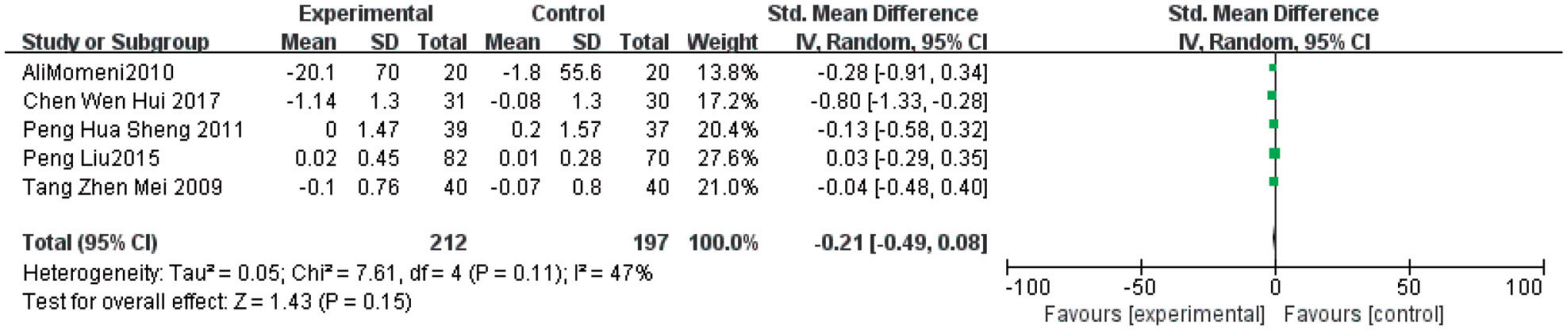
The meta-analysis results of Allopurinol for FBG.

**Figure 8.**
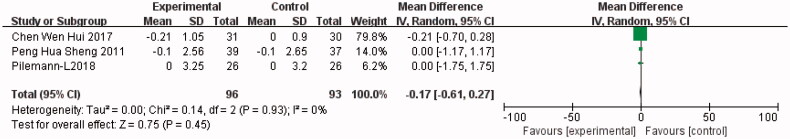
The meta-analysis results of Allopurinol for HbA1c.

**Figure 9.**
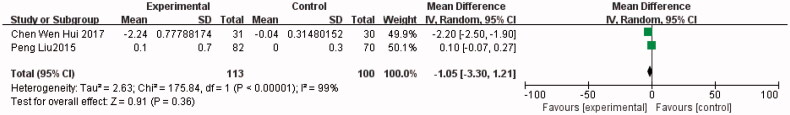
The meta-analysis results of Allopurinol for HOMA-IR.

Five trials [4,11,13,14,19] compared FBG between the allopurinol (*n* = 212) and control (*n* = 197) groups. As shown in Figure 7, FBG was lower in the allopurinol group than in the control group (SMD, −0.21; 95% CI, −0.49 to 0.08; *I*^2^, 47%; *p* = 0.15) (the Forest plots from Figures 7–9).

Three trials [11,12,19] compared HbA1c levels between the allopurinol (*n* = 96) and control (*n* = 93) groups. As shown in Figure 8, the HbA1c level was higher in the allopurinol group than in the control group (WMD, −0.17; 95% CI, −0.61 to 0.27; *I*^2^, 0%; *p* = 0.45).

Two trials [11,19] compared the HOMA-IR between the allopurinol (*n* = 113) and control (*n* = 100) groups. As shown in Figure 9, the HOMA-IR was lower in the allopurinol group than in the control group (WMD, −1.05; 95% CI, −3.30 to 1.21; *I*^2^, 99%; *p* = 0.36).

#### Changes in blood pressure

**Figure 10.**
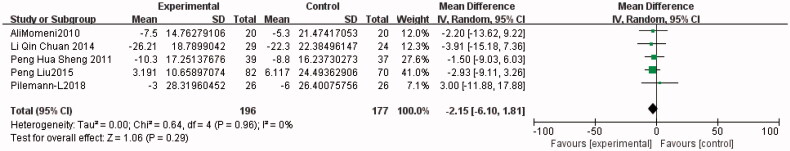
The meta-analysis results of Allopurinol for SBP.

**Figure 11.**
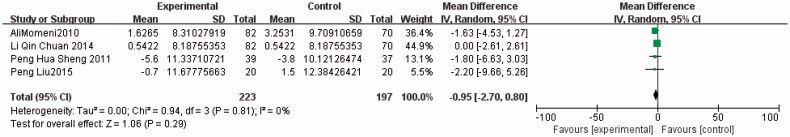
The meta-analysis results of Allopurinol for DBP.

Five trials [4,11–13,16] compared systolic blood pressure between the allopurinol (*n* = 196) and control (*n* = 177) groups. Lower systolic blood pressure was observed in the allopurinol group (WMD, −2.15; 95% CI, −6.10 to −1.81; *I*^2^, 0%; *p* = 0.29). Four trials [4,11,13,16] compared diastolic blood pressure between the allopurinol (*n* = 223) and control (*n* = 197) groups. As shown in Figure 11, DBP was lower in the allopurinol group than in the control group (WMD, −0.95; 95% CI, −2.70 to −0.80; *I*^2^, 0%; *p* = 0.29) (the Forest plots from Figures 10 and 11).

### GRADE for the outcomes

We evaluated all main outcome indicators using GRADEpro. The quality of evidence was downgraded for risk of bias, inconsistency, indirectness, imprecision, and publication bias. After a comprehensive analysis, the evidentiary body was formed, and it was found that one outcome indicator was of low quality and the other two outcome indicators were of moderate quality (GRADE.docx).

### Adverse effects

Three trials [11,17,19] assessed the safety of allopurinol. Safety was commonly assessed by evaluating serum levels of alanine aminotransferase (ALT) and aspartate aminotransferase (AST), the occurrence of pancytopenia, rash, gastrointestinal reactions, or any AEs related to allopurinol use. Some studies discovered AEs in the allopurinol group, including four cases [3,11,17] of diarrhea, five cases [11,17] of a decrease in AST and ALT levels, four cases [11,19] of vomiting, and two cases [17] of rash. Other studies did not discover any reported AEs.

## Discussion

Based on available clinical trials, our meta-analysis provided evidence for the efficacy and safety of allopurinol on the renal function of patients with diabetes. The results of this meta-analysis showed that compared with the control group, the levels of SUA [4,11–19] and 24H-P [4,13,17,18] in the allopurinol group were significantly lower. After a subgroup analysis of studies with a treatment duration of fewer than six months, we observed that the Scr level [4,11,13,15,16] of patients treated with allopurinol was significantly lower than that of the control group. However, after analyzing blood pressure [3,12–14,17] and the levels of glycemic parameters [4,11–19], no significant difference was observed. In addition, in terms of the safety of allopurinol, three trials [11,17,19] reported the AEs, including rash, diarrhea, vomiting, and changes in AST and ALT. However, no significant difference was observed between AEs.

Hyperuricemia is closely associated with gouty arthritis, metabolic syndrome, hypertension, cardiovascular diseases, and CKD, and is an independent risk factor for these diseases [5]. Allopurinol is the first-line therapy for patients who start urate-lowering therapy. Compared with febuxostat, allopurinol has higher cardiovascular safety and is strongly recommended for patients with moderate-to-severe CKD by the 2020 ACR guidelines [8]. There are many trials supporting our research results, which indicate that allopurinol can significantly reduce serum uric acid level, glycemic parameters, and blood pressure, and improve renal function in patients with type 1 or type 2 diabetes; these effects may be closely related to the dosage and treatment duration of allopurinol [5,11,13].

Tseng [3] found that the risk of urinary albumin-to-creatinine ratio increased by 1.183 times for every 1 mg/dL increase in serum uric acid level. Through the follow-up study of 1449 T2DM patients with normal renal function, Zoppini et al. [20] found that the incidence of diabetic nephropathy in a uric acid group was significantly higher than that in a normal uric acid group. Several articles [1,2,5,21] suggest that controlling blood uric acid levels can significantly protect early diabetic nephropathy and delay the progress of kidney disease, and serum uric acid level may be an independent risk factor for diabetic renal damage. Based on the above motivation, our study evaluated the efficacy and safety of allopurinol on renal function of patients with diabetes. The results showed that 24-h urinary protein was lower in the treatment group, which played a beneficial role in renal function, which is consistent with the results of many studies. Although some studies [1,2,5] have shown that long-term treatment with allopurinol can effectively control serum uric acid, reduce Scr, and protect the kidneys of patients with type 2 diabetes and asymptomatic hyperuricemia, we conducted a subgroup analysis on Scr in the treatment group and observed that the Scr of diabetes patients who received allopurinol treatment for less than six months was significantly lower than that of the control group. The reasons for the different results may be related to the long time span of administration reported in the literature and the small sample size of the studies included [9].

Chen et al. [5] analyzed four trials with a total of 314 patients; they found that ULT treatment with allopurinol did not significantly reduce HbA1c, but did effectively reduce blood glucose. This improvement was not observed in diabetes patients, which may be due to the fact that the correlation between uric acid level and FBG in diabetes patients changed from positive correlation to negative correlation compared with non-diabetes patients, and the influence of urate-lowering therapy on blood glucose seemed to be weakened. These findings are consistent with our results. Recent studies have shown [22] that the improvement of insulin resistance may be one of the mechanisms of FBG reduction in individuals receiving urate-lowering therapy. In addition, it has been confirmed that lowering uric acid can improve insulin resistance in obese mice. Previous studies [1,5] have found that allopurinol treatment can reduce HOMA-IR improve insulin resistance, and further delay the occurrence and development of microalbuminuria, thereby protecting the renal function of T2DM patients. However, although our study showed that HOMA-IR was lower in the allopurinol group than in the control group, the result was not significant, which may be related to the different degrees of damage to the islet function.

Hypertension is an important risk factor for kidney injury in T2DM patients caused by hyperuricemia. Hyperuricemia may activate the renin-angiotensin system (RAS), inhibit NO release, and lead to an increase in systemic vascular resistance [23]. Then, afferent arteriolar disease mediated by serum uric acid may occur, which would promote the development of hypertension. Kanbay et al. [24] found that allopurinol treatment improved the endothelial function of asymptomatic hyperuricemia patients and reduced blood pressure. It has also been shown [11,24] that, compared with conventional treatment, allopurinol treatment can reduce systolic and diastolic blood pressure levels in asymptomatic hyperuricemia patients with T2DM by maintaining the serum uric acid concentration below 360 mol/L. As our results showed, compared with the control group, blood pressure in the allopurinol group decreased, but the result was not statistically significant.

The strengths of this review include a comprehensive search, accurate inclusion criteria, careful consideration of study quality, and correct analytical approaches. We analyzed detailed relevant outcomes including changes in SUA, Scr, 24H-P, FBG, HOMA-IR, HbA1c, and blood pressure, as well as AEs. However, there were also some limitations of our study. First, the number of the included studies was small. We found only 10 relevant RCTs. Second, the sample size of the included trials varied greatly and the follow-up time in the 10 studies had large variations ranging from 2 to 36 months. Third, the heterogeneity of some indicators in this paper was too high. The reason for the high heterogeneity might be statistical heterogeneity, so we tried to use a random-effects model instead of a fixed-effects model. The other two reasons were clinical heterogeneity and methodological heterogeneity; they are the limitations of this study because it was difficult to carry out a satisfactory subgroup analysis given that the number of the included records was too small. Moreover, the differences in allopurinol dosage, regimen, and follow-up time may have contributed to heterogeneity.

## Conclusions

Our meta-analysis of RCTs showed that after oral administration of allopurinol, SUA levels were effectively decreased and renal function was protected in patients with diabetes. More RCTs with larger sample sizes and higher quality are needed to clarify the role of allopurinol in decreasing blood pressure, maintaining blood glucose levels, and improving renal function in patients with diabetes.

## Supplementary Material

Supplemental Material

## Abbreviations

DBP: diastolic blood pressure
RCTs: randomized controlled trials
SBP: systolic blood pressure
SUA: serum uric acid
FBG: fasting blood glucose

## References

[1] Liu P, Wang H, Zhang F, et al. The effects of allopurinol on the carotid intima-media thickness in patients with type 2 diabetes and asymptomatic hyperuricemia: a three-year randomized parallel-controlled study. Intern Med. 2015;54(17):2129–2137. 26328636 10.2169/internalmedicine.54.4310

[2] Richette P, Perez-Ruiz F, Doherty M, et al. Improving cardiovascular and renal outcomes in gout: what should we target? Nat Rev Rheumatol. 2014;10(11):654–661. 25136785 10.1038/nrrheum.2014.124

[3] Tseng CH. Correlation of uric acid and urinary albumin excretion rate in patients with type 2 diabetes mellitus in Taiwan. Kidney Int. 2005;68(2):796–801. 16014058 10.1111/j.1523-1755.2005.00459.x

[4] Peng HS, Xiao XR, Xu TY, et al. Effect of allopurinol on renal function in elderly patients with early diabetic nephropathy complicated with hyperuricemia. Clin Focus. 2011;26:1630–1631.

[5] Chen J, Ge J, Zha M, et al. Effects of uric acid-lowering treatment on glycemia: a systematic review and meta-analysis. Front Endocrinol. 2020;11:577. 10.3389/fendo.2020.00577 PMC7493655 33013687

[6] De Cosmo S, Viazzi F, Pacilli A, et al. AMD-Annals study group. Serum uric acid and risk of CKD in type 2 diabetes. CJASN. 2015;10(11):1921–1929. 26342044 10.2215/CJN.03140315 PMC4633786

[7] Srivastava A, Kaze AD, McMullan CJ, et al. Uric acid and the risks of kidney failure and death in individuals with CKD. Am J Kidney Dis. 2018;71(3):362–370. Mar 29132945 10.1053/j.ajkd.2017.08.017 PMC5828916

[8] Cohen RE, Pillinger MH, Toprover M. Something old, something new: the ACR gout treatment guideline and its evolution from 2012 to 2020. Curr Rheumatol Rep. 2020;23(1):4. 33245444 10.1007/s11926-020-00967-8

[9] Lin TC, Hung LY, Chen YC, et al. Effects of febuxostat on renal function in patients with chronic kidney disease: a systematic review and meta-analysis. Medicine. 2019;98(29):e16311. Jul 31335677 10.1097/MD.0000000000016311 PMC6709169

[10] Weisman A, Tomlinson GA, Lipscombe LL, et al. Allopurinol and renal outcomes in adults with and without type 2 diabetes: a retrospective, population-based cohort study and propensity score analysis. Can J Diabetes. 2021;45(7):641–649.e4. 33714662 10.1016/j.jcjd.2021.01.005

[11] Liu P, Chen Y, Wang B, et al. Allopurinol treatment improves renal function in patients with type 2 diabetes and asymptomatic hyperuricemia: 3-year randomized parallel-controlled study. Clin Endocrinol. 2015;83(4):475–482. Oct 10.1111/cen.12673 25400252

[12] Pilemann-Lyberg S, Persson F, Frystyk J, et al. The effect of uric acid lowering treatment on albuminuria and renal function in type 1 diabetes: a randomized clinical trial. Diabet Med. 2018;35(3):392–393. 29315768 10.1111/dme.13577

[13] Momeni A, Shahidi S, Seirafian S, et al. Effect of allopurinol in decreasing proteinuria in type 2 diabetic patients. Iran J Kidney Dis. 2010;4(2):128–132. 20404423

[14] Tang ZM. Intervention study on hyperuricemia and microalbuminuria in type 2 diabetes mellitus. Acta Medicinae Sinica. 2009;22:644–645.

[15] He LF, Yan SH, Li HS, et al. Effect of allopurinol on serum uric acid and urinary protein excretion excretion of diabetic nephropathy patients with hyperuricemia. J Hebei Medical University. 2018;39:649–652.

[16] Li QC. Effect of allopurinol on renal function in patients with diabetic nephropathy. Qingdao Med J. 2014;46:275–276.

[17] Tan Y, Fu JZ, Liang M, et al. Clinical observation of allopurinol in reducing proteinuria in patients with diabetic nephropathy. Med Innov China. 2011;8:15–16.

[18] Wang YY, Du HC, Du Y. Clinical observation of treatment to drop uric acid on diabetic nephropathy with symptomless hyperuricacidemia. Modern J Integr Traditional Chin West Med. 2013;20:2181–2183.

[19] Chen WH, Ding GH, Chen XH, et al. Effect of allopurinol on albuminuria in type 2 diabetic kidney disease with hyperuricemia. J Clin Nephrol. 2017;17:143–147.

[20] Zoppini G, Targher G, Chonchol M, et al. Serum uric acid levels and incident chronic kidney disease in patients with type 2 diabetes and preserved kidney function. Diabetes Care. 2012;35(1):99–104. 22028277 10.2337/dc11-1346 PMC3241303

[21] Kitada M, Kanasaki K, Koya D. Clinical therapeutic strategies for early stage of diabetic kidney disease. WJD. 2014;5(3):342–356. 24936255 10.4239/wjd.v5.i3.342 PMC4058738

[22] Baldwin W, McRae S, Marek G, et al. Hyperuricemia as a mediator of the proinflammatory endocrine imbalance in the adipose tissue in a murine model of the metabolic syndrome. Diabetes. 2011;60(4):1258–1269. 21346177 10.2337/db10-0916 PMC3064099

[23] Fathallah-Shaykh SA, Cramer MT. Uric acid and the kidney. Pediatr Nephrol. 2014;29(6):999–1008. 23824181 10.1007/s00467-013-2549-x

[24] Kanbay M, Huddam B, Azak A, et al. A randomized study of allopurinol on endothelial function and estimated glomular filtration rate in asymptomatic hyperuricemic subjects with normal renal function. Clin J Am Soc Nephrol. 2011;6(8):1887–1894. 21784838 10.2215/CJN.11451210 PMC3359530

