# Supplementary material for: Effects of allopurinol on renal function in patients with diabetes: a systematic review and meta-analysis
Source: Ren Fail. 2022 Jul 20;44(1):806–14. doi: 10.1080/0886022X.2022.2068443 (PMC9307109; doi:10.1080/0886022X.2022.2068443)
Supplement: Supplemental Material [file IRNF_A_2068443_SM5394.pdf]

Author(s):
Question: Allopurinol compared to Placebo or Conventional group for hyperuricemia in diabetes mellitus
Setting:
Bibliography: Allopurinol for hyperuricemia in diabetes mellitus. Cochrane Database of Systematic Reviews [Year], Issue [Issue].

Certainty assessment							№ of patients		Effect		Certainty	Importance
№ of studies	Study design	Risk of bias	Inconsistency	Indirectness	Imprecision	Other considerations	Allopurinol	Placebo or Conventional group	Relative (95% CI)	Absolute (95% CI)		
**24-hour urine protein**												
4	randomised trials	serious	not serious	not serious	not serious	none	158	150	-	MD **493.61 lower** (679.61 lower to 307.61 lower)	⨁⨁⨁◯ Moderate	
**subgroup analysis- Serum uric acid**												
10	randomised trials	serious	serious	not serious	not serious	none	446	420	-	SMD **2.32 lower** (3.29 lower to 1.35 lower)	⨁⨁◯◯ Low	
**subgroup analysis- Serum uric acid - Allopurinol vs Placebo**												
4	randomised trials	serious	serious	not serious	not serious	none	165	163	-	SMD **1.05 lower** (1.42 lower to 0.68 lower)	⨁⨁◯◯ Low	
**subgroup analysis- Serum uric acid - Allopurinol vs Conventional group**												
6	randomised trials	serious	serious	not serious	not serious	none	281	257	-	SMD **3.23 lower** (4.88 lower to 1.59 lower)	⨁⨁◯◯ Low	
**Serum creatinine**												
5	randomised trials	serious	not serious	not serious	not serious	none	250	231	-	SMD **0.45 lower** (0.96 lower to 0.06 higher)	⨁⨁⨁◯ Moderate	
**Serum creatinine - treatment duration ≥ 6 months**												
3	randomised trials	serious	not serious	not serious	not serious	none	191	174	-	SMD **0.51 lower** (1.33 lower to 0.32 higher)	⨁⨁⨁◯ Moderate	
**Serum creatinine - treatment duration ＜ 6 months**												
2	randomised trials	serious	not serious	not serious	not serious	none	59	57	-	SMD **0.41 lower** (0.77 lower to 0.04 lower)	⨁⨁⨁◯ Moderate	

CI: confidence interval; **MD:** mean difference; **SMD:** standardised mean difference
